# Rice-straw extract directly stimulates pre-symbiotic arbuscular mycorrhizal fungal growth and is associated with greater phosphorus acquisition in Sorghum

**DOI:** 10.3389/fpls.2026.1991797

**Published:** 2026-10-05

**Authors:** Ruiqian Li, Qiankun Zeng

**Affiliations:** 1 School of Life Sciences, Heilongjiang University, Harbin, China; 2 School of Chemistry and Molecular Engineering, Shanghai Key Laboratory for Urban Ecological Processes and Eco-Restoration, East China Normal University, Shanghai, China

**Keywords:** crop-residue-derived signals, fungal development, mycorrhizal phosphorus acquisition, plant–fungus interaction, pre-symbiotic activation

## Abstract

**Background:**

Improving arbuscular mycorrhizal fungal establishment may strengthen crop phosphorus acquisition and reduce dependence on phosphorus fertilizers. However, efficient symbiosis depends on fungal activation before root colonization, and practical approaches for stimulating this early stage remain limited.

**Methods:**

We tested whether aqueous rice-straw extract (RSE) directly activates AMF and improves subsequent symbiosis with sorghum. Spore germination, hyphal growth, root colonization, recoverable extraradical spore density, plant biomass, phosphorus acquisition, and symbiosis-related gene expression were quantified. Phenolic-depleted RSE was included to assess the contribution of phenolic compounds.

**Results:**

RSE showed a pronounced dose-dependent biphasic effect: low-to-moderate concentrations promoted AMF development, peaking at 0.3% with 74.1% spore germination, 18.48 mm hyphal length, and 27.5 branch points per spore, whereas 1.0% RSE markedly suppressed these responses. After 42 days, RSE increased root colonization from 22.5% to 51.3%, spore abundance from 58.4 to 106.4 per 50 g substrate, and extraradical hyphal length from 1.05 to 2.54 m per g. Shoot phosphorus uptake rose from about 2.3 to 5.9 mg per plant. Removing most phenolics markedly weakened these responses.

**Conclusion:**

RSE directly stimulated pre-symbiotic AMF development and was associated with greater subsequent colonization and phosphorus acquisition. Reduced activity after partial phenolic depletion suggests that phenolics contributed to the response, although other water-soluble components may also be involved.

## 1 Introduction

Phosphorus is indispensable for crop productivity, yet much of the phosphorus in soil is poorly mobile or strongly sorbed and therefore difficult for roots to acquire (Paz-Ares et al., 2022; Poirier et al., 2022). Arbuscular mycorrhizal fungi (AMF) offer a natural solution: their fine hyphae extend beyond the root surface and deliver phosphorus to host plants (Qin et al., 2022). Recent field and controlled studies have shown that AMF can improve phosphorus uptake and crop performance in maize and sorghum, although the benefit varies with soil conditions, crop genotype and agricultural management (Hou et al., 2021; Chandra et al., 2022). However, inoculation alone does not always lead to stable root colonization or effective symbiosis. Recent studies have shown that organic amendments, either alone or combined with inorganic phosphorus, can improve crop growth, soil carbon status and phosphorus availability (Khan et al., 2022; Khan et al., 2023b; Abbas et al., 2024). These effects are partly associated with changes in microbial biomass and soil carbon-to-phosphorus stoichiometry, which regulate phosphorus immobilization, mobilization and subsequent plant availability (Khan et al., 2023a; Khan et al., 2025). However, these studies have mainly examined soil-level nutrient processes rather than the direct responses of AMF to water-soluble compounds released from crop residues. A key question is therefore how to activate AMF early enough to improve reliable root colonization and nutrient exchange.

Before entering a root, AMF spores must germinate and produce hyphae that grow toward a suitable host (Liu et al., 2023). This pre-symbiotic stage is controlled by chemical communication. Flavonoids such as quercetin in root exudates can stimulate AMF germination and colonization, while differences in exuded chemical signals can change the strength of plant–AMF associations (Tian et al., 2021; Yu et al., 2022). Strigolactones also promote pre-symbiotic fungal growth, although fungal strains may respond differently (Klein et al., 2024). Recent molecular studies have further clarified fungal phosphate transport, root recognition, chitin-related signaling and the coordinated gene activity of plant and fungal partners (Xie et al., 2022; Serrano et al., 2024). Metabolomic studies show that phenylpropanoids and flavonoids are particularly dynamic during early AMF establishment (Yurkov et al., 2022). These studies highlight the importance of plant-derived chemical cues in regulating pre-symbiotic AMF development and suggest that similar signals may also be released from non-living plant materials. Recent evidence further indicates that phosphorus acquisition is governed by coordinated interactions among fungal hyphae, phosphorus-mobilizing bacteria and host roots, rather than by the plant–fungus partnership alone (Khan et al., 2026). This broader hyphosphere perspective also highlights the need to distinguish direct fungal stimulation from indirect effects mediated by surrounding microorganisms.

Research to date has mainly focused on root-derived signals and purified bioactive compounds, leaving the signaling potential of non-living plant materials largely unexplored. Crop residues are another abundant source of plant-derived compounds, yet their possible role as chemical cues for beneficial fungi is poorly explored. Rice straw contains a diverse pool of water-soluble bioactive compounds, including phenolic acids such as p-coumaric, ferulic, and caffeic acids, which have been consistently detected in rice-straw-derived fractions (Jumrus et al., 2022; Beladhadi et al., 2023; Silva et al., 2024). In sorghum, specialized root metabolites can also influence AMF colonization and phosphorus nutrition (Figueiredo de Oliveira et al., 2025), while changes in root-colonizing AMF communities are linked with plant growth and phosphorus responses (Frew and Aguilar-Trigueros, 2024). These findings raise an important question: can water-soluble compounds released from crop residues directly activate AMF, rather than acting only as nutrients or organic amendments?

To address this gap, we tested whether aqueous RSE can directly activate the pre-symbiotic development of AMF and examine whether RSE treatment was associated with greater subsequent root colonization and phosphorus acquisition. By separating the pre-symbiotic fungal stage from the later plant–fungus interaction, this reductionist design enabled us to test whether RSE acted directly on the fungus under controlled conditions. It was not intended to reproduce the full complexity of natural soil communities. AMF spores were first exposed to graded RSE concentrations without a host plant, allowing germination, hyphal elongation and branching to be measured directly. We then examined whether RSE treatment was also associated with greater root colonization, recoverable extraradical spore density, sorghum growth and phosphorus acquisition in separate plant experiments. Intact RSE was further compared with phenolic-depleted RSE and representative phenolic compounds to determine how much of the activity could be attributed to phenolics. This study moves beyond evaluating rice straw as a simple mycorrhizal amendment by asking whether crop-residue-derived chemical cues can influence AMF before root colonization and whether this response is associated with subsequent nutrient acquisition.

## 2 Materials and methods

### 2.1 Materials and experimental design

The experiment used *Rhizophagus intraradices* as the arbuscular mycorrhizal fungus (AMF) because it is a widely studied AMF species with reliable colonization under controlled conditions. Sorghum was selected as the host because it is an agriculturally important cereal that responds to AMF colonization, particularly under low-phosphorus conditions. The AM fungus Rhizophagus intraradice (Nicol. & Gerd.) Walker & Schüßler (isolate RI-HLJ01) was obtained from the Institute of Plant Nutrition and Resources, Beijing Academy of Agriculture and Forestry Sciences, China. Sorghum [Sorghum bicolor (L.) Moench] cultivar Longza 11 was provided by Heilongjiang Academy of Agricultural Sciences, Harbin, China. Seeds were surface-sterilized with 70% ethanol for 1 min, rinsed with sterile water, treated with 2% NaClO for 5 min, and rinsed five times with sterile water. Spore viability was assessed on 2% water agar after 7 days at 28 °C in darkness; spores with germ tubes longer than the spore diameter were considered viable. Seedlings were initially grown in a static hydroponic system using 2-L opaque plastic containers (three plants per container, supported by foam discs) filled with one-quarter-strength low-phosphorus Hoagland solution (pH 6.0), which was renewed every 3 days. For the pot experiments, seedlings were transplanted into pots containing the growth substrate. The growth substrate consisted of a sterilized sand–vermiculite mixture (1:1, v/v), autoclaved at 121 °C for 30 min on two consecutive days. The hydroponic stage was used only for seedling establishment, and no AMF inoculum was added during this stage. Roots became colonized only after transplantation, when 500 viable spores were placed in the substrate near the roots. Inoculum viability exceeded 85% in all experiments. All experiments were conducted in a plant growth chamber at the School of Life Sciences, Heilongjiang University, Harbin, China, under a 12 h light/12 h dark photoperiod, 25/20 °C day/night temperature, 60–70% relative humidity, and 300 μmol m^−2^ s^−1^ light intensity. The study comprised three sequential experiments: (1) a 10-day host-free assay examining the direct effects of RSE on AMF spore germination and hyphal development; (2) a 28-day pot experiment selecting effective concentrations of RSE, quercetin and vanillic acid; and (3) a 42-day pot experiment comparing intact RSE, phenolic-depleted RSE and reference compounds. The pot, rather than each individual plant, was considered the independent experimental unit. All plant-treatment groups were inoculated with AMF because the plant experiments were designed to compare how RSE and reference compounds modified an established AMF–sorghum system, rather than to quantify sorghum mycorrhizal dependency. The experimental groups and sample numbers are shown in **Table 1**.

**Table 1:** Overview of the experimental design, groups, and measurements.

Experiment	Groups and concentrations	Replication	Duration	Main measurements
RSE test on AMF spores	RSE at 0, 0.1, 0.3, 0.5, 0.7, and 1.0% (v/v)	6 replicates; 30 spores per replicate	10 days	Germination, hyphal length, branch points
Testing different concentrations in sorghum	CK; GR24 0.1 µM; RSE 0.1–1.0%; quercetin 0.01–1.0 µM; vanillic acid 0.01–1.0 µM	6 pots per group	28 days	Total/arbuscule colonization, shoot dry mass
42-day pot experiment	CK; GR24; RSE 0.3%; RSE - PD; quercetin 0.1 µM; vanillic acid 0.01 µM; quercetin + vanillic acid	8 pots per group	42 days	Colonization structures, spores, extraradical hyphae, biomass, P uptake, fungal DNA, RT-qPCR

CK, one-quarter-strength low-phosphorus Hoagland solution; GR24, synthetic strigolactone analogue; RSE, rice-straw extract; RSE-PD, rice-straw extract depleted of most phenolic compounds; AMF, arbuscular mycorrhizal fungi; RT-qPCR, reverse-transcription quantitative polymerase chain reaction.

### 2.2 Preparation of rice-straw extract and dephenolization

Air-dried rice straw was ground into a fine powder (200–500 μm). The powder was mixed with ultrapure water at a 1:10 (w/v) ratio and extracted at 60 °C for 2 h with continuous mixing. The mixture was then centrifuged at 8,000 × g for 15 min. The liquid portion was collected, passed through 0.45- and 0.22-μm filters, and stored at −20 °C for no longer than four weeks. Before use, the extract was diluted with low-phosphate Hoagland solution to final RSE concentrations of 0.1, 0.3, 0.5, 0.7 and 1.0% (v/v). RSE concentration was defined as the volume of undiluted RSE stock per final volume of treatment solution; thus, 0.3% (v/v) RSE contained 3 mL of stock per liter of final solution. These concentrations were selected to cover low, intermediate and relatively high exposure levels and to permit detection of a potentially nonlinear concentration–response relationship. The same concentration series was used in the host-free spore assay and the preliminary sorghum experiment, with 0% RSE serving as the control. To determine the role of phenolic compounds in RSE, we prepared another RSE sample in which most phenolics were removed. The RSE stock was mixed with 10% (w/v) insoluble polyvinylpolypyrrolidone (PVPP) for 30 min at 4 °C, followed by centrifugation and sterile filtration. This procedure removed approximately 82% of total phenolics while retaining 94% of soluble sugars. Based on the stock total phenolic content of 94.9 mg GAE L^−1^, the residual total phenolic content in the RSE-PD stock was approximately 17.1 mg GAE L^−1^ (18% of the original), corresponding to about 51.2 μg GAE L^−1^ in the 0.3% RSE-PD treatment. This phenolic-depleted extract was referred to as RSE-PD. Quercetin and vanillic acid were prepared as 1 mM stock solutions in dimethyl sulfoxide (DMSO).

### 2.3 Measurement of the main compounds in rice-straw extract

For each RSE batch, pH, total phenolic content (TPC), total flavonoid content (TFC), soluble sugar content, and eight individual phenolic compounds were measured. pH was determined with a calibrated pH meter at 25 °C. TPC was measured by the Folin–Ciocalteu method: 0.5 mL sample + 2.5 mL 10% Folin reagent, then 2 mL 7.5% Na_2_CO_3_, incubated 30 min in darkness at 25 °C, absorbance at 765 nm. TFC was measured by the AlCl_3_ colorimetric method: 1 mL sample + 0.3 mL 5% NaNO_2_ (6 min), 0.3 mL 10% AlCl_3_ (6 min), 4 mL 1 M NaOH, diluted to 10 mL, absorbance at 510 nm. Soluble sugars were measured by the anthrone–sulfuric acid method: 0.5 mL sample + 2.5 mL anthrone reagent, boiling water bath 10 min, absorbance at 620 nm. Absorbance was recorded on a Shimadzu UV-1800 spectrophotometer. Gallic acid (0–100 mg L^−1^), quercetin (0–50 mg L^−1^), and glucose (0–200 mg L^−1^) were used as standards for TPC, TFC, and soluble sugars, respectively. Calibration curves were constructed with six levels in triplicate (R (Paz-Ares et al., 2022) > 0.998). LOD/LOQ were 0.85/2.58 mg GAE L^−1^ for TPC, 0.42/1.27 mg QE L^−1^ for TFC, and 2.1/6.4 mg L^−1^ for soluble sugars. Blanks, triplicates, and spiked recovery tests (97.6–102.4% for TPC, 96.8–103.1% for TFC, 98.1–101.7% for sugars; RSD < 3.5%) were included.

Individual phenolic compounds (vanillic acid, p-coumaric acid, ferulic acid, syringic acid, protocatechuic acid, caffeic acid, quercetin, and rutin) were quantified by UHPLC-MS/MS using an Agilent 1290 Infinity UHPLC coupled to an Agilent 6460 triple-quadrupole mass spectrometer with an ESI source (Agilent Technologies, Santa Clara, CA, USA). Separation was performed on an Agilent ZORBAX Eclipse Plus C18 column (2.1 × 100 mm, 1.8 μm) at 30 °C. The mobile phase consisted of 0.1% formic acid in water (A) and acetonitrile (B) at 0.3 mL min^−1^, with a 2 μL injection volume. The gradient was: 0–1 min, 5% B; 1–8 min, 5–95% B; 8–10 min, 95% B; 10–10.1 min, 95–5% B; 10.1–12 min, 5% B. MS detection was in negative ESI mode with multiple reaction monitoring (MRM); transitions were optimized for each compound. Source parameters were: capillary voltage 3500 V, nebulizer pressure 35 psi, drying gas temperature 300 °C, drying gas flow 10 L min^−1^. No internal standard was used; external calibration was applied. Standards were prepared in methanol/water (50:50, v/v) at six levels (0.05–20 mg L^−1^) for each compound, with linear ranges and R^2^ > 0.998. LOD and LOQ were 0.005–0.015 and 0.015–0.045 mg L^−1^, respectively. QC procedures included method blanks, calibration checks every 10 samples, duplicate injections, and spiked recovery tests; recoveries were 92.5–105.3% with RSDs < 4.8%, and matrix effects ranged from 88.2% to 107.6%. Data were acquired and processed using Agilent MassHunter Workstation software (B.10.00). Each compound was measured separately in the three RSE batches, and the coefficient of variation (CV) was calculated to compare batches. Total phenolics and flavonoids were expressed as gallic acid equivalents (GAE) and quercetin equivalents (QE), respectively, and soluble sugars as glucose equivalents; these values represent assay responses relative to the selected standards, not summed individual compound concentrations. To allow direct comparison with the reference compound treatments, the final concentrations in the 0.3% (v/v) RSE treatment were calculated from the stock values. The total phenolic concentration was approximately 284.7 μg GAE L^−1^, and the concentrations of individual phenolics were 24.8 μg L^−1^ for vanillic acid, 19.4 μg L^−1^ for p-coumaric acid, 17.6 μg L^−1^ for ferulic acid, 15.2 μg L^−1^ for syringic acid, 10.9 μg L^−1^ for protocatechuic acid, 8.6 μg L^−1^ for caffeic acid, 6.2 μg L^−1^ for quercetin, and 10.6 μg L^−1^ for rutin. In comparison, the quercetin treatment (0.1 μM) corresponded to 30.2 μg L^−1^, and the vanillic acid treatment (0.01 μM) corresponded to 1.68 μg L^−1^. Thus, the individual phenolic concentrations in 0.3% RSE were within the same order of magnitude as the reference treatments, although the ratios differed among compounds. These concentrations allowed comparison with the reference treatments, although their environmental relevance in field soils remains to be established.

### 2.4 Test the direct effect of RSE on AMF spores

To determine whether RSE could directly stimulate AMF before contact with plant roots, spores were surface-sterilized with 70% ethanol for 30 s and 2% NaClO for 2 min, then rinsed five times with sterile water. Surface-disinfected AMF spores were grown on 2% water agar containing 0, 0.1, 0.3, 0.5, 0.7 or 1.0% (v/v) RSE. For each RSE concentration, 180 spores were tested and divided into six groups of 30 spores. The spores were kept in the dark at 28 °C for 10 days. Germination was observed after 10 days at 28 °C in darkness; a spore was considered germinated when the germ tube exceeded the spore diameter. Hyphal length and branch points were measured under a microscope using image analysis.

### 2.5 Selection of RSE, quercetin and vanillic acid concentrations

A 28-day pot experiment was used to determine which concentration of RSE, quercetin and vanillic acid gave the best response in sorghum. The experiment included a low-phosphate negative control (CK), 0.1 μM GR24 as a positive control, five RSE concentrations (0.1, 0.3, 0.5, 0.7 and 1.0%), and four concentrations each of quercetin and vanillic acid (0.01, 0.1, 0.5 and 1.0 μM). Six pots were used for each group, and the pot positions were randomly assigned. Treatments were applied at 50 mL per pot every 3 days. All groups received the same volume of solution, and pot positions were rearranged every 7 days to minimize positional effects within the growth chamber. Each pot contained three sorghum plants and was inoculated with 500 viable AMF spores at transplantation. After 28 days, the plants were harvested. AMF root colonization and shoot and root dry mass were measured. For RSE, quercetin and vanillic acid, the concentration that produced the highest root colonization without reducing shoot growth was selected for the following 42-day experiment.

### 2.6 Comparison of RSE with reference compounds

A 42-day pot experiment was conducted to confirm the effects of the selected RSE concentration and to compare it with several reference groups. The experiment included CK, GR24 (0.1 μM), RSE (0.3%), RSE with most phenolic compounds removed (RSE-PD), quercetin (0.1 μM), vanillic acid (0.01 μM), and a mixture of quercetin and vanillic acid at the same concentrations. Eight pots were used for each group, with two sorghum plants in each pot. Except for the specified treatment compounds, all groups received identical substrate, nutrient solution, cultivation conditions and solution volumes. Pots were randomly assigned to treatments and periodically repositioned within the growth chamber. After 42 days, the plants and growth substrate were collected. We measured AMF root colonization, fungal spore number, and the length of fungal hyphae growing outside the roots. Shoot phosphorus uptake was also measured to determine whether the increase in AMF was accompanied by better phosphorus acquisition. We also measured the amount of AMF in the roots and the expression of genes related to AMF colonization and phosphorus transport.

### 2.7 Measurement of AMF colonization, spore and hyphal growth, sorghum growth and phosphorus uptake

AMF growth was measured both inside and outside sorghum roots. Roots were cleared in 10% KOH at 90 °C for 30 min, acidified in 1% HCl, stained with 0.05% trypan blue, and destained. Colonization was scored by the gridline intersect method (200 intersections per sample). Colonization scoring was performed blindly, with sample identities coded before microscopic examination. The stained roots were examined under a light microscope to determine the percentage of roots containing fungal hyphae, arbuscules and vesicles. Recoverable extraradical AMF spores were isolated from 50 g of thoroughly mixed growth substrate by wet sieving and sucrose-density centrifugation and then counted under a stereomicroscope. Spores within colonized roots were not included. Because the inoculated spores were not labelled, residual inoculated spores could not be distinguished reliably from spores formed during the experiment; therefore, this measurement was interpreted as extraradical spore density rather than new sporulation. Fungal hyphae outside the roots were collected by membrane filtration and their length was measured under a microscope.

Plant growth was measured from root length and dry mass. Root systems were placed in a transparent tray and scanned using a root imaging system, and total root length was calculated with root-analysis software. Shoots and roots were dried at 65 °C until their weight remained unchanged and then weighed separately. Shoot phosphorus concentration was measured by inductively coupled plasma optical emission spectrometry (ICP–OES) after acid digestion. Phosphorus uptake per plant was calculated from shoot phosphorus concentration and shoot dry mass.

### 2.8 Measurement of AMF in roots and related genes

To measure the amount of AMF in sorghum roots, DNA was extracted from 100 mg fresh root tissue using a DNeasy Plant Mini Kit. The AMF gene RiEF1α was used to estimate fungal abundance, and a sorghum reference gene was used to correct for differences in the amount of root DNA among samples. Gene expression was also measured to examine changes related to AMF colonization and phosphorus transport. RNA was extracted from the roots and converted to cDNA before RT-qPCR analysis. Five sorghum genes (SbD14L, SbCCaMK, SbRAM1, SbPT11 and SbSTR2) and the fungal phosphorus transporter gene RiPT were measured. These genes represent AMF recognition, root colonization, arbuscule formation and phosphorus transport. Relative gene expression was calculated using the comparative Ct (2^−ΔΔCt^) method, with the CK group set to 1. RNA was extracted using TRIzol reagent, treated with DNase I, and reverse-transcribed using PrimeScript RT reagent Kit. qPCR was performed on an ABI 7500 system with SYBR Green Master Mix in 10-μL reactions. Thermal conditions were 95 °C for 30 s, followed by 40 cycles of 95 °C for 5 s and 60 °C for 34 s. Melt curves were generated from 60 to 95 °C; a single peak was observed for each primer pair. Primer efficiencies were 92–105%, with R^2^ > 0.99. SbActin and RiEF1α were used as reference genes for sorghum and fungal transcripts, respectively. Three technical replicates and three biological replicates were analyzed. No genome-wide sequencing, transcriptomic profiling or untargeted metabolomic analysis was performed.

### 2.9 Data analysis and statistics

Data are presented as mean ± standard error (SE). Each experiment was analyzed separately. Differences among experimental groups were tested by one-way analysis of variance (ANOVA). When ANOVA showed a significant difference, Tukey’s test was used to determine which groups differed from each other. For the RSE concentration experiment, a quadratic regression was also used to examine whether the measured values increased at low concentrations but decreased at higher concentrations. The relationship between AMF root colonization and plant growth was tested using Pearson correlation. For RT-qPCR, statistical tests were performed using ΔCt values, whereas relative expression calculated by the comparative Ct (2^−ΔΔCt) method was used for figures and reporting. Data were checked for normal distribution and similar variation among groups before ANOVA. For all ANOVAs, exact F, degrees of freedom, and P values are reported in the text. Tukey’s HSD was used for multiple comparisons. Effect sizes are reported as partial eta-squared (η^2^p). For the colonization–biomass relationship, Pearson correlation was first used, and partial correlation controlling for treatment group was then performed to avoid pooling treatment effects. Statistical significance was set at P < 0.05.

## 3 Results

### 3.1 Characterization of RSE and evaluation of early AMF growth

The composition of RSE was similar across the three independently prepared batches (**Table 2**). The undiluted RSE stock contained 94.9 ± 3.6 mg GAE L^−1^ of total phenolics, and vanillic acid was the most abundant phenolic compound measured (8.28 ± 0.46 mg L^−1^). Variation among batches was small, with CV values below 6% for all measured phenolic compounds.

**Table 2:** Main components of RSE across three batches.

Analyte	Unit	Mean ± SD	CV (%)
Total phenolic content	mg GAE L^−1^	94.9 ± 3.6	3.8
Total flavonoid content	mg QE L^−1^	25.3 ± 0.2	0.8
Soluble sugars	g glucose equivalents L^−1^	1.74 ± 0.08	4.6
pH	—	5.63 ± 0.09	1.6
Vanillic acid	mg L^−1^	8.28 ± 0.46	5.6
p-Coumaric acid	mg L^−1^	6.47 ± 0.28	4.3
Ferulic acid	mg L^−1^	5.85 ± 0.29	5.0
Syringic acid	mg L^−1^	5.08 ± 0.25	4.9
Protocatechuic acid	mg L^−1^	3.63 ± 0.10	2.8
Caffeic acid	mg L^−1^	2.88 ± 0.14	4.9
Quercetin	mg L^−1^	2.06 ± 0.11	5.3
Rutin	mg L^−1^	3.53 ± 0.19	5.4

Values are presented as mean ± SD from three independently prepared RSE batches. RSE, rice-straw extract; SD, standard deviation; GAE, gallic acid equivalents; QE, quercetin equivalents; CV, coefficient of variation; UHPLC–MS/MS, ultra-high-performance liquid chromatography–tandem mass spectrometry. Total phenolic and flavonoid contents are expressed relative to gallic acid and quercetin standards, respectively, and do not represent the summed concentrations of individual compounds. Individual phenolic compounds were quantified separately by UHPLC–MS/MS and are reported in mg L^−1^. All values refer to the undiluted RSE stock and are not expressed per gram of dry straw or as concentrations in the final diluted treatment solutions.

The early growth of AMF varied with RSE concentration (**Figures 1A, B**). The clearest effect occurred at 0.3% RSE, where spore germination reached 74.1%, hyphal length reached 18.48 mm, and branch points reached 27.5 per spore. All three measures differed significantly among RSE concentrations (P < 0.001). At 1.0% RSE, germination decreased to 39.2%, hyphal length to 6.69 mm, and branch points to 8.9 per spore, indicating that increasing the RSE concentration beyond the optimal level weakened its stimulatory effect and reduced spore germination, hyphal growth and branching.

**Figure 1:**
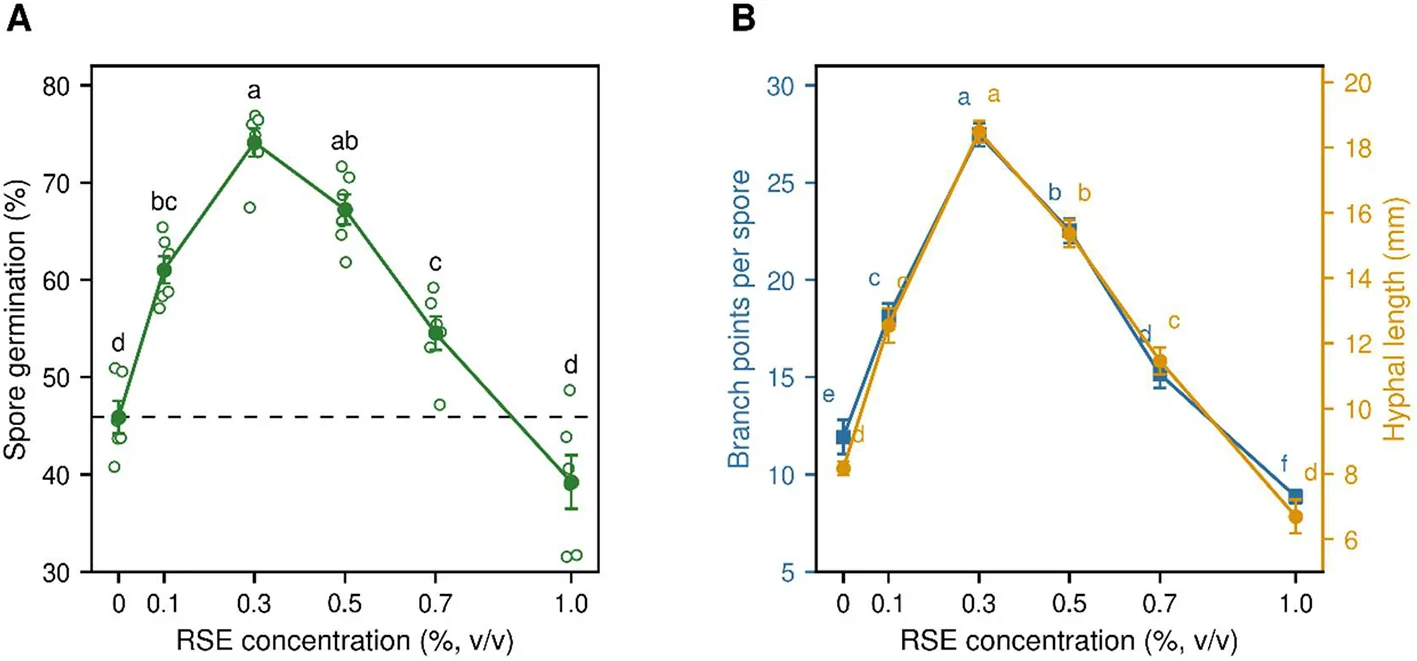
Effects of rice-straw extract (RSE) concentration on the early development of arbuscular mycorrhizal fungi (AMF). **(A)** Percentage of AMF spores germinating after 10 days. The dashed horizontal line indicates the mean germination under the 0% RSE control. **(B)** Number of branch points per germinated spore (blue) and hyphal length (orange) after 10 days. Values are means ± standard errors (SE; n = 6 independent Petri dishes). Different lowercase letters indicate significant differences among RSE concentrations for the same response variable according to one-way analysis of variance followed by Tukey’s honestly significant difference *post hoc* test (*P* < 0.05). RSE concentrations are expressed as percentage volume/volume (%, v/v); mm, millimetres.

### 3.2 Effects of different concentrations of RSE, quercetin and vanillic acid on AMF colonization

CK served as the untreated control, while GR24 was included as a positive control for AMF colonization. RSE was most effective at 0.3%, increasing total colonization from 16.1% in CK to 38.4% (Tukey-adjusted P < 0.05); this value did not differ significantly from the 43.5% observed with GR24 (P > 0.05; **Figure 2A**). Higher RSE concentrations reduced both total and arbuscule colonization. Quercetin showed its strongest effect at 0.1 μM, reaching 40.5% total colonization and 12.3% arbuscule colonization, both significantly higher than CK (Tukey-adjusted P < 0.05), whereas vanillic acid was most effective at 0.01 μM (35.9% total colonization) (**Figures 2B, C**). Total colonization was positively related to shoot dry mass (r = 0.89, P < 0.001; **Figure 2D**), with greater arbuscule formation mainly observed in highly colonized plants. These results supported the concentrations selected for the 42-day experiment.

**Figure 2:**
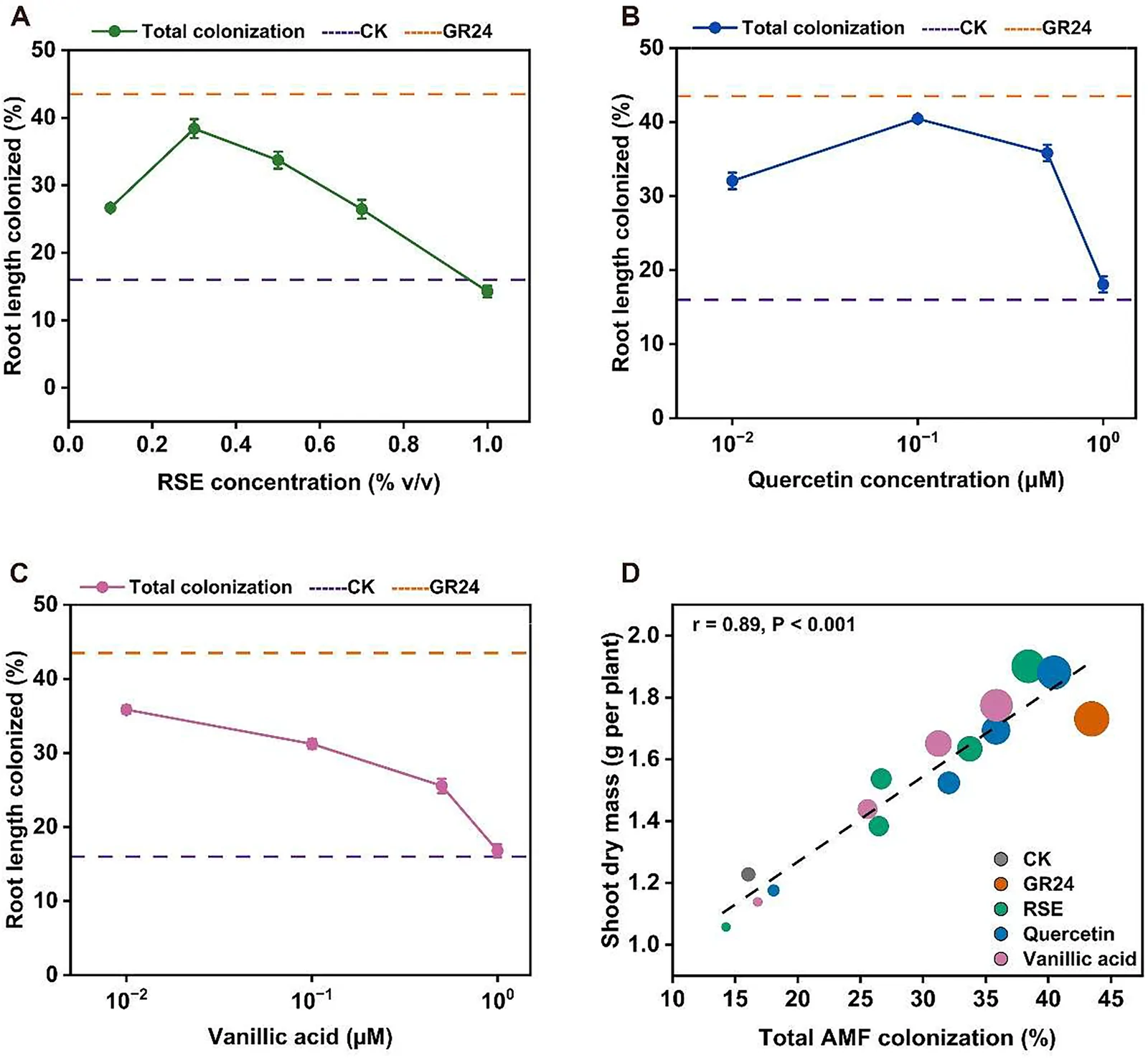
Effects of RSE, quercetin and vanillic acid concentrations on AMF colonization and sorghum growth. **(A)** Total root colonization at different RSE concentrations. **(B)** Total colonization at different quercetin concentrations. **(C)** Total colonization at different vanillic acid concentrations. **(D)** Relationship between total AMF colonization and shoot dry mass across experimental groups; partial correlation controlling for treatment group is shown; bubble size represents arbuscule colonization.

### 3.3 Effects of RSE and phenolic compounds on AMF colonization, spore number and extraradical hyphal length

After 42 days, AMF colonization, spore number and hyphal growth were compared among the experimental groups. One-way ANOVA showed significant treatment effects on total colonization (F(6,49) = 18.6, P < 0.001, η^2^p = 0.69), spore density (F(6,49) = 25.4, P < 0.001, η^2^p = 0.76), and extraradical hyphal length (F(6,49) = 31.2, P < 0.001, η^2^p = 0.79). RSE differed from CK after Tukey adjustment (P < 0.05). RSE increased total root colonization from 22.5% in CK to 51.3% (Tukey-adjusted *P* < 0.05), with no significant difference from GR24 or quercetin (both *P* > 0.05; **Figure 3A**). The three fungal structures in the roots showed similar increases but differed in their relative amounts (**Figure 3B**). Intraradical hyphae were the most abundant fungal structure in the roots and increased clearly with RSE, GR24 and quercetin compared with CK. Arbuscule colonization increased from about 5% in CK to 17.2% with RSE and 17.8% with quercetin. Vesicle colonization increased with RSE and GR24. Quercetin and vanillic acid also increased vesicle colonization, while the increase with quercetin plus vanillic acid was similar, whereas the increase was smaller with RSE-PD. Recoverable extraradical spore density was higher with RSE than with CK (106.4 versus 58.4 spores per 50 g substrate; Tukey-adjusted *P* < 0.05; **Figure 3C**). This measurement included potentially residual inoculated spores and therefore should not be interpreted as newly formed spores alone. Extraradical hyphal length increased from 1.05 m g^−1^ in CK to 2.54 m g^−1^ with RSE (Tukey-adjusted *P* < 0.05; **Figure 3D**). Compared with intact RSE, RSE-PD showed 33.8% lower total colonization, 25.1% fewer spores and 36.1% less extraradical hyphal length.

**Figure 3:**
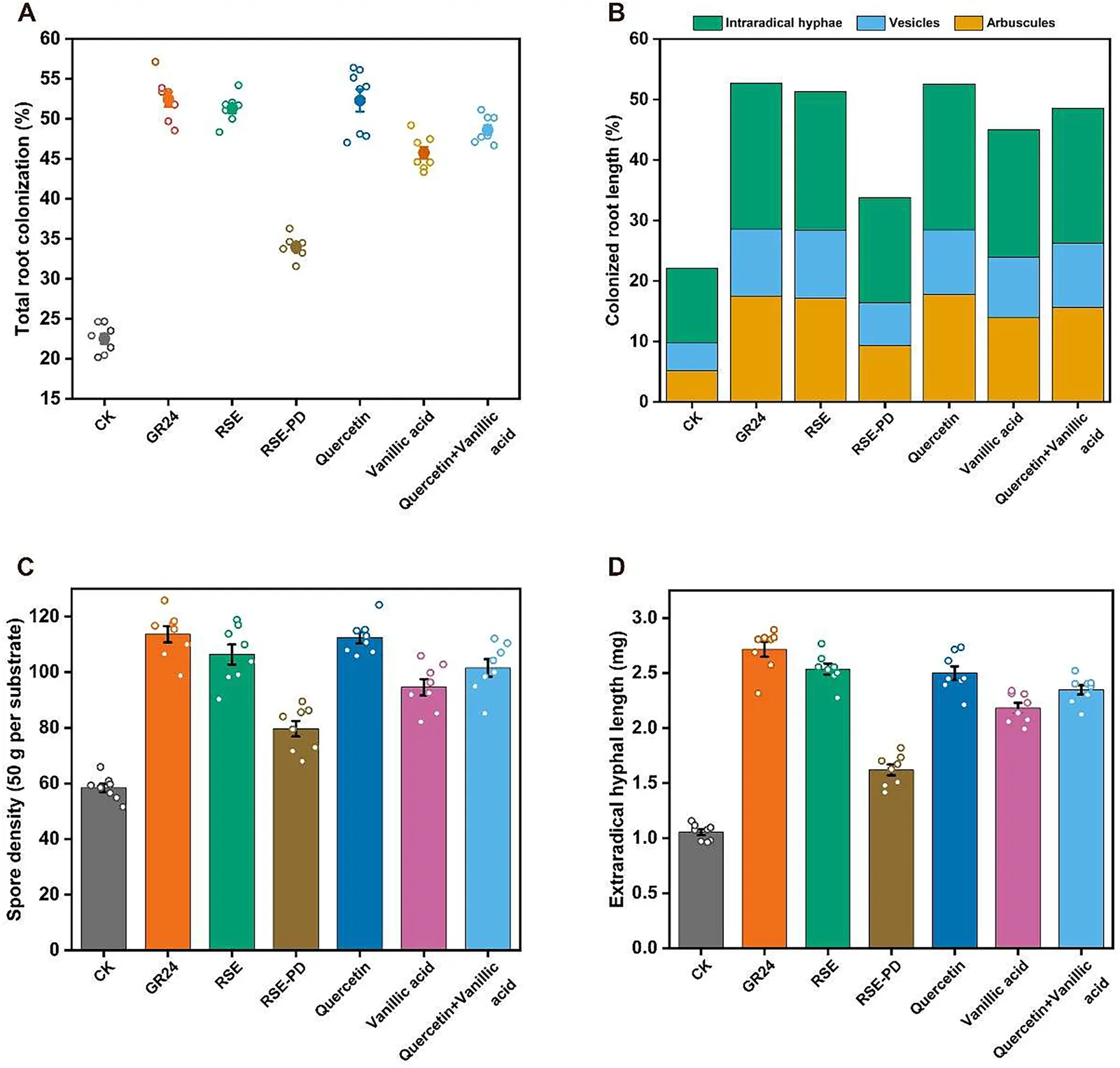
Effects of RSE and phenolic compounds on AMF growth after 42 days. **(A)** Total AMF colonization in sorghum roots. **(B)** Colonization by intraradical hyphae, arbuscules and vesicles. **(C)** AMF spore number per 50 g of growth substrate. **(D)** AMF hyphal length outside the roots per gram of dry substrate.

### 3.4 Sorghum growth, phosphorus uptake, and AMF-associated gene expression

Because RSE increased AMF growth, we next examined whether these changes were accompanied by better sorghum growth and phosphorus uptake. After 42 days, RSE-treated plants showed the greatest shoot biomass, with shoot dry mass increasing from 1.36 g per plant in the CK group to 2.19 g per plant (**Figure 4A**). RSE treatment also increased root dry mass compared with CK (Tukey-adjusted *P* < 0.05). Shoot dry mass also increased following treatment with GR24, quercetin, vanillic acid, or their quercetin–vanillic acid combination, but the increases were smaller than that observed with RSE. Shoot phosphorus uptake reached 5.9 mg per plant with RSE, compared with approximately 2.3 mg per plant in CK (Tukey-adjusted *P* < 0.05; **Figure 4B**). Shoot dry mass and P uptake also differed significantly among treatments (shoot dry mass: F(6,49) = 14.8, P < 0.001, η^2^p = 0.64; P uptake: F(6,49) = 22.7, P < 0.001, η^2^p = 0.74). RSE also increased the expression of genes related to AMF colonization and phosphorus transport (**Figure 4C**). Compared with CK, RSE increased the expression of SbD14L, SbCCaMK, SbRAM1, SbPT11, SbSTR2 and RiPT by 3.9, 3.5, 5.5, 6.2, 4.9 and 4.5-fold, respectively (Tukey-adjusted P < 0.05 for all). RSE-PD induced smaller increases (1.7–2.4-fold), which were lower than intact RSE (P < 0.05). **Figure 4D** summarizes the relative changes in five key parameters compared with CK. Within each experimental group, the five colored bars represent total root colonization, spore number, extraradical hyphal length, shoot dry mass and shoot phosphorus uptake. RSE showed clear increases in all five measurements, whereas the increases with RSE-PD were consistently smaller. The weaker responses observed after phenolic depletion indicate that phenolic compounds contributed substantially to the effects of RSE on both AMF development and sorghum growth.

**Figure 4:**
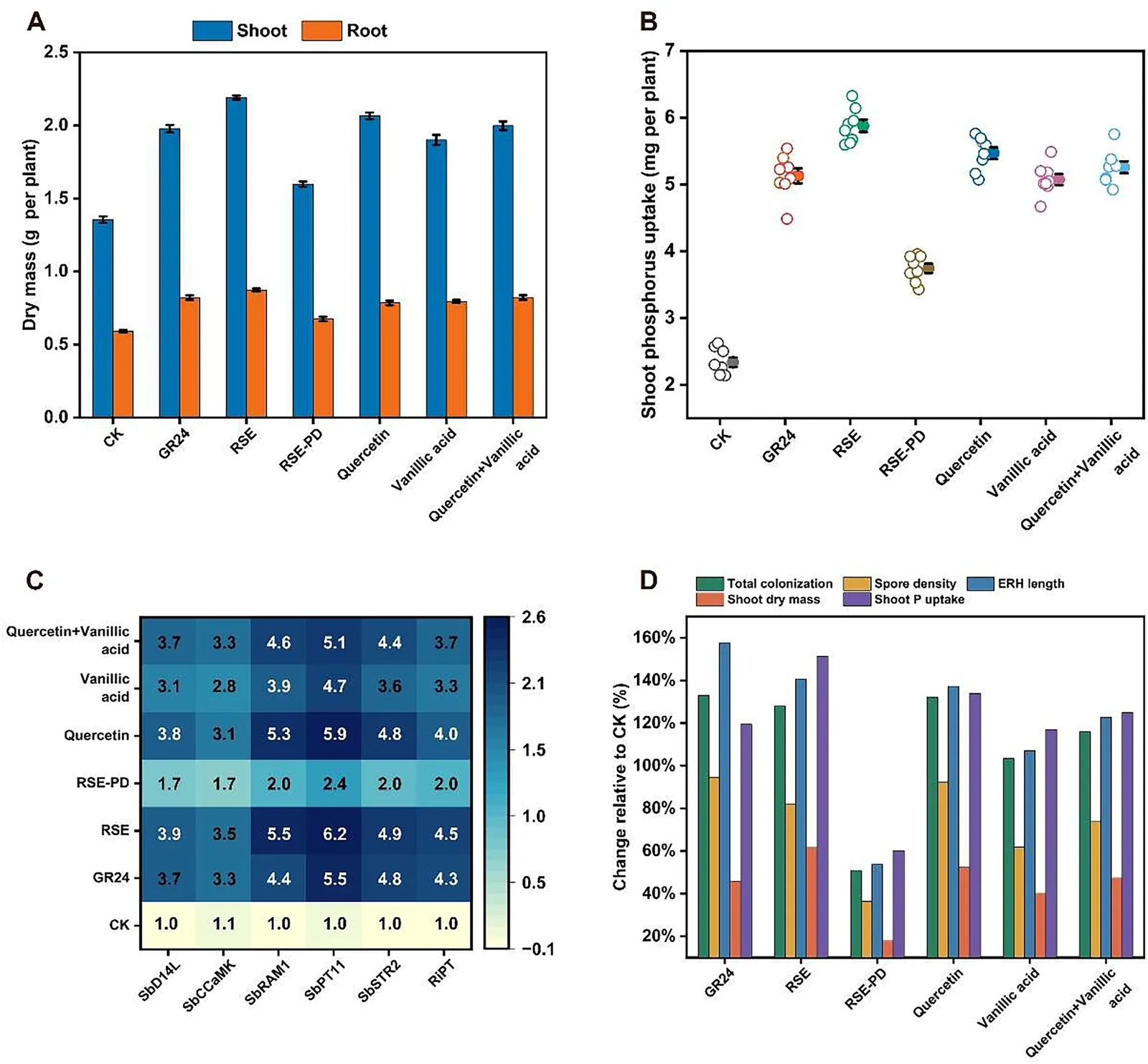
Effects of RSE and phenolic compounds on sorghum growth, phosphorus uptake and AMF-related gene expression. **(A)** Shoot and root dry mass in the seven experimental groups. **(B)** Shoot phosphorus uptake. **(C)** Relative expression of sorghum genes related to AMF colonization and phosphorus transport (SbD14L, SbCCaMK, SbRAM1, SbPT11 and SbSTR2) and the fungal phosphorus transporter gene RiPT. **(D)** Percentage change relative to CK in total root colonization, spore density, extraradical hyphal (ERH) length, shoot dry mass and shoot phosphorus uptake.

## 4 Discussion

### 4.1 Rice-straw extract directly stimulates pre-symbiotic AMF development in a host-free assay

A key finding was that RSE directly stimulated AMF germination and hyphal development in the absence of a host plant, with the strongest response at an intermediate concentration. Because these responses were measured in a host-free system, they cannot be explained by changes in sorghum growth or root metabolism. The simplest interpretation is that water-extractable compounds in rice straw can act directly on the fungus during its early development. This idea is consistent with recent evidence that AMF can respond to small plant-derived molecules before root contact. For example, monoterpene glucosides produced by plant roots can directly stimulate hyphal branching in Rhizophagus species (Tominaga et al., 2023), while strigolactones function as conserved rhizosphere signals that promote AMF symbiosis (Kodama et al., 2022). Mycorrhizal signals can also modify host development before colonization; for example, chitin tetramer promotes rice root development through LysM receptor-like kinases (He et al., 2024). However, this plant-mediated response differs from the direct fungal response to RSE observed in our host-free assay. Our results extend this idea beyond compounds released by living roots and suggest that crop residues may also contain molecules capable of modifying the pre-symbiotic behaviour of AMF. As AMF are obligate biotrophs, the host-free assay represents only a transient pre-symbiotic stage and cannot support completion of the fungal life cycle. RSE may contain necromass-derived signals or nutrients that stimulate exploratory hyphal growth, rather than signals that specifically indicate the presence of a living host. Although RSE treatment was associated with greater sorghum-root colonization in the separate pot experiments, the present design did not track individual fungi from pre-symbiotic activation to root colonization. Whether such stimulation affects host location or partner recognition in natural soil therefore remains unknown.

### 4.2 The effect of RSE depends strongly on concentration

Increasing the RSE concentration did not produce a progressively stronger fungal response. Instead, the response peaked at an intermediate concentration and declined at the highest concentration tested. This clear rise-and-fall response suggests that RSE has an effective concentration range rather than a simple dose-dependent nutritional effect. At moderate concentrations, stimulatory compounds may reach levels that favour fungal growth, whereas at higher concentrations some components may become inhibitory or disturb the balance among active compounds. This feature is important if RSE is eventually developed as a practical treatment: an excessive dose may reduce rather than improve its effectiveness. However, our experiments do not identify the compounds responsible for the decline at high RSE concentrations. Measurements of individual components across the concentration gradient, followed by testing of isolated fractions, will be needed to explain why the fungal response changes above the optimum.

### 4.3 Phenolics are important, but they are not the whole explanation

The comparison between intact RSE and phenolic-depleted RSE helps narrow down the chemical basis of the response. Removing approximately 82% of total phenolics reduced total colonization by 33.8%, spore abundance by 25.1%, and extraradical hyphal length by 36.1% relative to intact RSE. This loss of activity supports an important contribution from phenolic compounds. However, phenolic-depleted RSE remained active, and neither quercetin nor vanillic acid, alone or together, reproduced the full effect of RSE. The active fraction is therefore unlikely to be explained by a single compound. This is biologically plausible because AMF can respond to chemically different classes of plant metabolites (Lidoy et al., 2023). However, the current experiment cannot determine whether interactions among RSE components are additive, synergistic or antagonistic. Only quercetin and vanillic acid were tested individually and in one fixed combination, and their combined effect did not reproduce the full activity of intact RSE. Moreover, PVPP treatment removed multiple phenolics simultaneously and may also have altered other compounds. Future studies should combine chemical fractionation with concentration-matched factorial and add-back experiments to identify active compounds and quantify their individual and interactive effects on AMF development and mycorrhization.

Several limitations of the chemical characterization should be noted. We did not measure minerals, amino acids, or other water-soluble nutrients in RSE, so a nutritional contribution to the observed fungal stimulation cannot be fully excluded. Although soluble sugars were measured (1.74 g L^−1^ in stock, corresponding to 5.2 mg L^−1^ in 0.3% RSE), other nutrients may also be present. In addition, PVPP removed only about 82% of total phenolics, leaving a residual phenolic pool that may still contribute to the remaining activity of RSE-PD. Stronger depletion using multiple sorbents or repeated treatments would help clarify the role of residual phenolics. Finally, only eight individual phenolic compounds were quantified, and functional validation was limited to quercetin and vanillic acid; testing additional compounds and fractions at environmentally relevant concentrations is needed to identify the full active principle.

### 4.4 Greater fungal development is associated with improved phosphorus acquisition

Across the separate experiments, direct pre-symbiotic fungal stimulation and greater root colonization, extraradical fungal development and shoot phosphorus uptake were observed under RSE treatment. However, these parallel findings do not establish that early fungal stimulation caused the subsequent plant responses. The increased expression of SbRAM1, SbPT11 and RiPT was also consistent with stronger AM colonization and P exchange. Plant adaptation to low-phosphate conditions involves coordinated changes in root architecture, phosphate transport, root exudation and plant–microbe interactions, providing a broader crop-improvement context for the association between AMF development and phosphorus acquisition (Hu et al., 2025). Recent work in maize similarly shows that functional AM symbiosis can improve plant phosphate status and alter the surrounding microbial environment (Lu et al., 2023). Recent studies further indicate that organic carbon inputs and soil C/P stoichiometry can regulate microbial phosphorus immobilization and mobilization, thereby changing phosphorus availability to plants (Khan et al., 2022; Khan et al., 2023b; Khan et al., 2023a; Khan et al., 2025). These soil-level processes may have contributed to the phosphorus response observed in the present study, although they are distinct from the direct fungal stimulation demonstrated in the host-free assay. These results support a link between greater fungal development and improved phosphorus acquisition, but they should not be interpreted as proof that RSE increases plant phosphorus uptake only through AMF. Our pot experiment did not include corresponding non-mycorrhizal groups. Therefore, the study cannot quantify sorghum mycorrhizal dependency or determine whether the increases in biomass and phosphorus uptake were specifically mediated by AMF. Direct effects of RSE on root growth, phosphorus availability, nutrient supply or other microorganisms cannot be excluded. A factorial experiment combining RSE presence or absence with AMF inoculation or non-inoculation will be required to separate these effects. Likewise, changes in gene expression show association rather than direct molecular regulation by RSE. Future experiments using AMF-free controls and isotope-labelled P would help separate fungal and plant contributions to the observed P gain.

### 4.5 Biological significance, limitations and next steps

The broader value of these findings lies in viewing crop residues as more than sources of carbon and nutrients. Their soluble chemical components may influence beneficial microorganisms before a stable plant–microbe association is formed. This could eventually complement AMF inoculation by improving the conditions under which fungal propagules establish symbiosis. Several limitations, however, need to be resolved before this idea can be generalized. Only one AMF isolate and one sorghum cultivar were tested, although AMF isolates can differ substantially in colonization, plant growth promotion and nutrient acquisition. Natural soils contain minerals, organic matter and diverse microorganisms that may adsorb, degrade or transform RSE compounds. During straw decomposition, microorganisms may convert the original RSE compounds into metabolites with different biological activities. Their concentrations in soil solution may also vary with soil pH, texture, mineral composition, organic matter, moisture and microbial activity. Therefore, the nominal RSE concentrations used under controlled conditions may not correspond directly to fungal exposure in field soils. The selected 0.3% RSE concentration also cannot be converted directly into an agronomic straw-return rate because extraction efficiency, soil water volume, compound adsorption and decomposition differ among field conditions. The present study therefore does not define how much rice straw or RSE should be applied per unit of soil or field area. Field experiments should relate realistic straw-return rates to measured concentrations of active compounds in soil solution before practical recommendations are made. Future studies should measure the persistence and transformation of RSE compounds in contrasting soils and relate their actual soil-solution concentrations to AMF responses. Moreover, a single root system may interact with multiple AMF taxa and other microorganisms, including mycorrhizal helper bacteria. More broadly, plants and diverse rhizosphere microorganisms form complex interaction networks that regulate nutrient cycling, crop performance and agricultural sustainability (Shi et al., 2024). Recent evidence indicates that interactions among AMF hyphae, phosphorus-mobilizing bacteria and plant roots jointly regulate phosphorus transformation and transfer in the hyphosphere (Khan et al., 2026). Consequently, microbial communities may alter both the chemical composition of RSE and the fungal response observed under simplified conditions. These interactions may alter fungal activation and root colonization; therefore, the responses observed in our single-fungus system require validation in more complex microbial communities. Field responses to AMF are known to vary with fungal identity, plant group, soil properties and climate. Finally, the active chemical fraction of RSE remains unresolved. Testing multiple AMF genotypes, crop varieties and soils, together with chemical fractionation and field validation, will therefore be essential. Validation under realistic straw-return rates and field conditions is required before RSE can be considered for managing mycorrhizal establishment.

## 5 Conclusion

This study shows that rice-straw extract can directly stimulate the pre-symbiotic development of AMF under host-free conditions. In separate pot experiments, RSE treatment was associated with greater mycorrhizal development and phosphorus acquisition in sorghum, but the absence of non-mycorrhizal controls prevents these plant responses from being attributed specifically to AMF. Reduced activity after partial phenolic depletion suggests a contribution from phenolics, although residual phenolics and other water-soluble components may also be involved. Validation across different soils, microbial communities, crop cultivars, AMF taxa and realistic straw-return rates is required before field application can be considered.

## Data Availability

The raw data supporting the conclusions of this article will be made available by the authors, without undue reservation.
